# Weak Interactions between *Salmonella enterica* FlhB and Other Flagellar Export Apparatus Proteins Govern Type III Secretion Dynamics

**DOI:** 10.1371/journal.pone.0134884

**Published:** 2015-08-05

**Authors:** Jonathan L. McMurry, Tohru Minamino, Yukio Furukawa, Joshua W. Francis, Stephanie A. Hill, Katy A. Helms, Keiichi Namba

**Affiliations:** 1 Department of Molecular & Cellular Biology, Kennesaw State University, Kennesaw, Georgia, United States of America; 2 Graduate School of Frontier Biosciences, Osaka University, Osaka, Japan; University of Osnabrueck, GERMANY

## Abstract

The bacterial flagellum contains its own type III secretion apparatus that coordinates protein export with assembly at the distal end. While many interactions among export apparatus proteins have been reported, few have been examined with respect to the differential affinities and dynamic relationships that must govern the mechanism of export. FlhB, an integral membrane protein, plays critical roles in both export and the substrate specificity switching that occurs upon hook completion. Reported herein is the quantitative characterization of interactions between the cytoplasmic domain of FlhB (FlhB_C_) and other export apparatus proteins including FliK, FlhA_C_ and FliI. FliK and FlhA_C_ bound with micromolar affinity. K_D_ for FliI binding in the absence of ATP was 84 nM. ATP-induced oligomerization of FliI induced kinetic changes, stimulating fast-on, fast-off binding and lowering affinity. Full length FlhB purified under solubilizing, nondenaturing conditions formed a stable dimer via its transmembrane domain and stably bound FliH. Together, the present results support the previously hypothesized central role of FlhB and elucidate the dynamics of protein-protein interactions in type III secretion.

## Introduction

The bacterial flagellum is a proton-driven rotary nanomachine responsible for motility in many species [1,2,3]. Most proteins that comprise a flagellum reside beyond the cytoplasmic membrane and must be secreted. Secretion occurs via a specialized type III secretion system (T3SS or “export apparatus”[4,5]). Utilizing protonmotive force [6,7,8], the apparatus translocates flagellar proteins across the cytoplasmic membrane into the central channel within the growing flagellum through which they transit to their final location [9]. Homologous T3SSs effect many modes of bacterial pathogenesis using needle-like structures that closely resemble flagella [10].

The core flagellar T3SS consists of three soluble proteins (FliH, FliI and FliJ) and six integral membrane proteins (FlhA, FlhB, FliO, FliP, FliQ and FliR) that are housed within the membrane-supramembrane ring of the basal body. Like most of the other proteins, FlhB is necessary for secretion and is critical for the substrate specificity switching that occurs upon hook completion as the export apparatus shifts from rod and hook-type proteins to filament-type proteins [4,11]. FlhB undergoes asparagine-mediated autohydrolysis at N269-P270 [12,13]. Mutations in FlhB that slow or abolish this cleavage give rise to dramatically altered flagellar structures due to defects in switching [14]. FliK is one of the rod- and hook-type substrates recognized via the flagellar T3SS during hook assembly [15]. A specific interaction of FlhB with FliK is central to the switch, in which the T3SS stops exporting rod and hook-type proteins and begins exporting filament-type proteins [16,17] via a mechanism modeled as a “temporal tape measure” in which FliK interacts with both FlhB and hook proteins [18,19]. The interaction between FliK and FlhB is thought to vary as a function of hook length, though the details of how remain unknown [20]. In addition to FliK, FlhB has been reported to bind FliH, FliI, FliJ and perhaps the cytoplasmic domain of FlhA (FlhA_C_) [21], though the veracity and consequences of these interactions are largely unknown. Full-length FlhA and FlhB exhibited no binding to each other in affinity blots [22].

While a great deal of work has described apparatus proteins with respect to requirements for secretion, qualitative interactions and structure, understanding of dynamic interactions has lagged. Kinetic relationships are fertile ground for exploration and characterizing them will provide a better understanding of T3S and transmembrane transport in general. Much of what is known about interactions among export proteins, substrates and chaperones derives from copurification and affinity blotting experiments that have limitations such as requirements for attainment of equilibrium and that only high affinity interactions can be observed. Using a type of optical biosensing, biolayer interferometry (BLI)[23], and analytical ultracentrifugation, the present study was able to address oligomerization of full-length, membrane integrated FlhB as well as the complex kinetic interactions of FlhB and its cytoplasmic domain with other T3S apparatus proteins.

Similar to surface plasmon resonance (SPR), BLI allows real-time measurement of protein-protein interactions and determination of kinetic and affinity constants [24]. Ligand proteins are tethered to fiber optic sensors and dipped into analyte-containing buffers to measure association. Dissociation is monitored after movement to buffer without analyte. Instrument response, measured in nanometers of shift of the interference pattern of white light caused by analyte-induced changes in the distance between two reflecting surfaces over time, yields association and dissociation rate information. Fits of raw data to kinetic models allow assignment of rate and affinity constants. In the case of simple binding, fits to single exponentials allow determination of k_off_ from the dissociation phase since reassociation is negligible due to dilution of dissociated analyte. Fitting the association phase yields observed rate constants (k_obs_), from which k_on_ can be extracted given analyte concentration and k_off_.

We were able to characterize the mostly weak, complex interactions of FlhB_C_ with FliK, FlhA_C_ and FliI. Provision of ATP to FliI dramatically altered binding, weakening affinity. FlhB was shown to form a stable dimer via the transmembrane domain and to bind FliH. The current work not only sheds light on dynamic events in flagellar T3S, but also sets a foundation for future studies utilizing the membrane proteins of the apparatus in optical biosensing.

## Materials and Methods

### Overexpression and purification

Plasmids used in this study are shown in S1 Table. His-tagged variants of the soluble export proteins and the cytoplasmic domains of FlhA and FlhB (“FlhA_C_” and “FlhB_C_”) were overproduced and purified. Overnight cultures of *E*. *coli* BL21DE3(pLysS) cells harboring plasmids encoding His-tagged proteins were subcultured and grown in Luria broth at 30°C to an OD_600_ ~ 0.4. Expression was induced by addition of 0.2 mM IPTG, after which growth was continued for four hours. Cells were harvested by centrifugation and pellets were frozen at -80°C until use.

All purification steps were performed on ice or at 4°C. Pellets from 1 L cultures were thawed and resuspended in 25 ml lysis buffer (50 mM Tris pH 8.0, 500 mM NaCl, 10 mM imidazole, 0.1% Tween-20 and 200 μg ml^-1^ lysozyme). Resuspended cells were passed through a French press at 20,000 psi and then subjected to centrifugation for 20 min at 10,000 x G at 4°C. The resulting clarified supernatant was transferred to a tube containing 1 ml of equilibrated Talon (BD Biosciences) immobilized metal affinity chromatography (IMAC) resin.

Batch binding was allowed to proceed with gentle agitation for 20 min after which the resin was pelleted by brief centrifugation and washed twice with 20 ml wash buffer (50 mM Tris pH 8.0, 500 mM NaCl, 25 mM imidazole, 0.1% Tween-20). The resin was transferred to a column and washed with an additional 10 ml. Elution was achieved by addition of elution buffer (wash buffer with 250 mM imidazole). Proteins were exchanged into HBS-T (10 mM HEPES, pH 7.4, 150 mM NaCl. 0.05% Tween 20) by gel filtration and used immediately, or glycerol was added to 10% and proteins were snap frozen in liquid nitrogen and stored at -80°C until use. Concentrations were determined by Bradford assay [25] using BSA as standard.

Full-length FlhB was overproduced and purified under nondenaturing conditions from solubilized crude membrane fractions as described for FlhA [22]. For AUC studies the uncleavable variant of full-length FlhB, FlhB(N269A), the method of Fleming et al. [26] was modified as follows: cells overexpressing FlhB(N269A) were resuspended in 10 mM phosphate buffer pH 8.0, 500 mM NaCl, 20% glycerol, 10 mM β-mercaptoethanol, 10 mM imidazole and lysed by sonication. Lysates were centrifuged at 10,000 x g to pellet unbroken cells. Supernatants were ultracentrifuged at 100,000 x g to pellet membranes. The crude membrane fraction was resuspended in lysis buffer (same as above but with 1% Thesit), homogenized and stirred at 4°C for 1 hour. After centrifugation at 100,000 x g for 45 min, the supernatant was retained as solubilized membrane fraction and subjected to IMAC to purify the FlhB(N269A). Wash and elution buffers were the same as the lysis except containing 20mM and 250 mM imidazole, respectively. To exchange the Thesit for E_8_C_5_, a detergent with the same partial specific volume as water and hence amenable to analytical ultracentrifugation, purified FlhB(N269A), ~25 ml, was diluted in 1 L dilution buffer (10 mM phosphate buffer, pH 8.0, 1% Thesit, 20% glycerol, 20mM β-mercaptoethanol and then loaded onto a 1 mL SP Sepharose column. The column was washed with 50 ml of 10 mM phosphate buffer, 33 mM C_8_E_5_, 10 mM NaCl at 1 ml min^-1^. FlhB(N269A) was eluted in 1 ml fractions in the phosphate/C_8_E_5_ buffer with 500 mM NaCl.

### Optical biosensing

All biolayer interferometry (BLI) measurements were made on a FortéBio (Menlo Park, CA) Octet QK biosensor using streptavidin (“SA”) sensors. Assays were performed in 96-well microplates at 25°C. All volumes were 200 μL. Ligand proteins were exchanged into HBS-T by passage over a desalting column. Biotinylation by amine crosslinking to NHS-LC-LC-biotin (succinimidyl-6-[biotinamido]-6-hexanamidohexanoate) was performed at a 5:1 molar ratio of biotin to protein for 30 min at room temperature followed by separation of protein from free biotin by repeated passage over a desalting column. After loading ligands onto SA sensors, a baseline was established in buffer prior to association at varying analyte concentrations. Dissociation was subsequently measured in buffer only. All phases were done in HBS-T, except the full-length FlhB experiment, in which the Tween was replaced with 1% Triton X-100. Raw data were analyzed with GraphPad Prism.

For numerical simulation of FliK-FlhB_C_ binding, a conformational change model (A + B ⇌ AB ⇌ AB*) was made in which A is analyte (FlhB_C_), B is ligand, AB is the bound complex and AB* is a conformationally altered state. Rate constants k_1_ and k_2_ govern association and dissociation of the free proteins and k_3_ and k_4_ describe the shift to and from the AB* state, respectively. The set of differential equations used for the simulations were:

$$\begin{array}{c} dAB\;/\;dt\;=\;k_{1}A•B\;+\;k_{4}AB*\;-\;k_{2}AB\;-\;k_{3}AB*\\ dA\;/\;dt\;=\;k_{2}AB\;-\;k_{1}A•B\\ dAB*\;/\;dt\;=\;k_{3}AB\;-\;k_{4}AB* \end{array}$$

Data were plotted as fractions of maximal binding (B_max_), which was iteratively determined. A 15% correction factor to account for differences between the signal produced by AB* relative to AB was included in the simulations.

### Analytical ultracentrifugation

Sedimentation equilibrium ultracentrifugation was performed using a Beckman Optima XL-A ultracentrifuge and an AnTi 60 rotor essentially as described [27], except that the buffer contained 33 mM C_8_E_5_. Scans were collected at 280 nm with a spacing of 0.001 cm in the step mode with twenty averages per step. Three scans were superimposed prior to analysis with Optima XL-A/XLI version 4.0 (Beckman).

## Results

### Biosensing Survey

**Fig 1:**
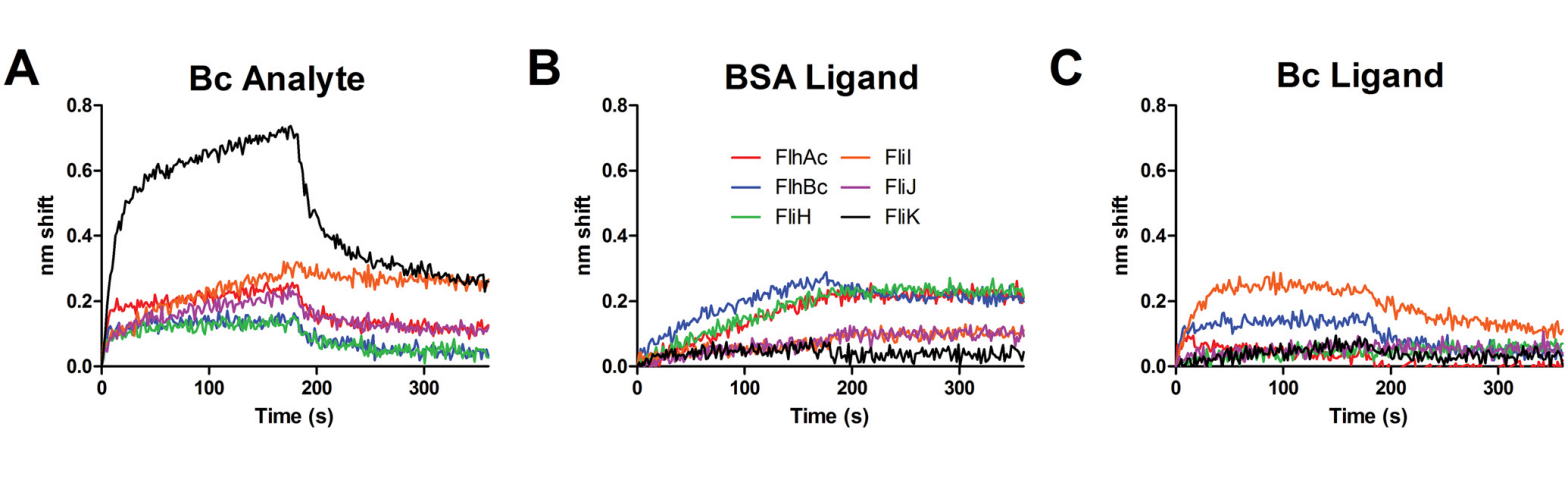
FlhB_C_ kinetic survey. Purified T3S proteins were biotinylated and used as ligands. All analyte concentrations were 1 μM. Association and dissociation phases were 180 seconds in all cases. A, FlhB_C_ analyte binding to ligands FlhA_C_ (red), FlhB_C_ (blue), FliH (green), FliI (orange), FliJ (magenta) and FliK (black). B, nonspecific binding control of analyte apparatus proteins binding to sensor-tethered biotinylated BSA. Analyte colors are the same as ligand colors in A. C, Analyte apparatus proteins binding to ligand FlhB_C_. Colors are the same as in B.

To examine dynamic interactions between FlhB_C_ and other apparatus proteins, FlhB_C_ was used as analyte versus each of the other apparatus proteins as ligand (Fig 1A). A starkly different shift profile from nonspecific control binding (Fig 1B, black trace) was observed for FliK. Smaller differences were noted for all other export proteins, e.g. a small amplitude fast on state for FlhA_C_, indicating some interaction with FlhB_C_. Observed binding was complex and nonspecific binding (NSB) as evidenced by response to BSA as ligand was in many cases significant. FliJ, FliH and FlhB_C_ ligands also exhibited binding different from BSA, but were resistant to further analysis due to NSB, low signal and other reasons. Biotinylated FlhB_C_ was tethered to SA sensors and screened for binding versus analyte soluble export apparatus proteins at 1 μM (Fig 1C). Differences in FliI binding were noted. FliK-FlhB_C_, FlhA_C_-FlhB_C_ and FlhB_C_-FliI interactions were selected for further kinetic characterization.

### Kinetic characterizations

**Fig 2:**
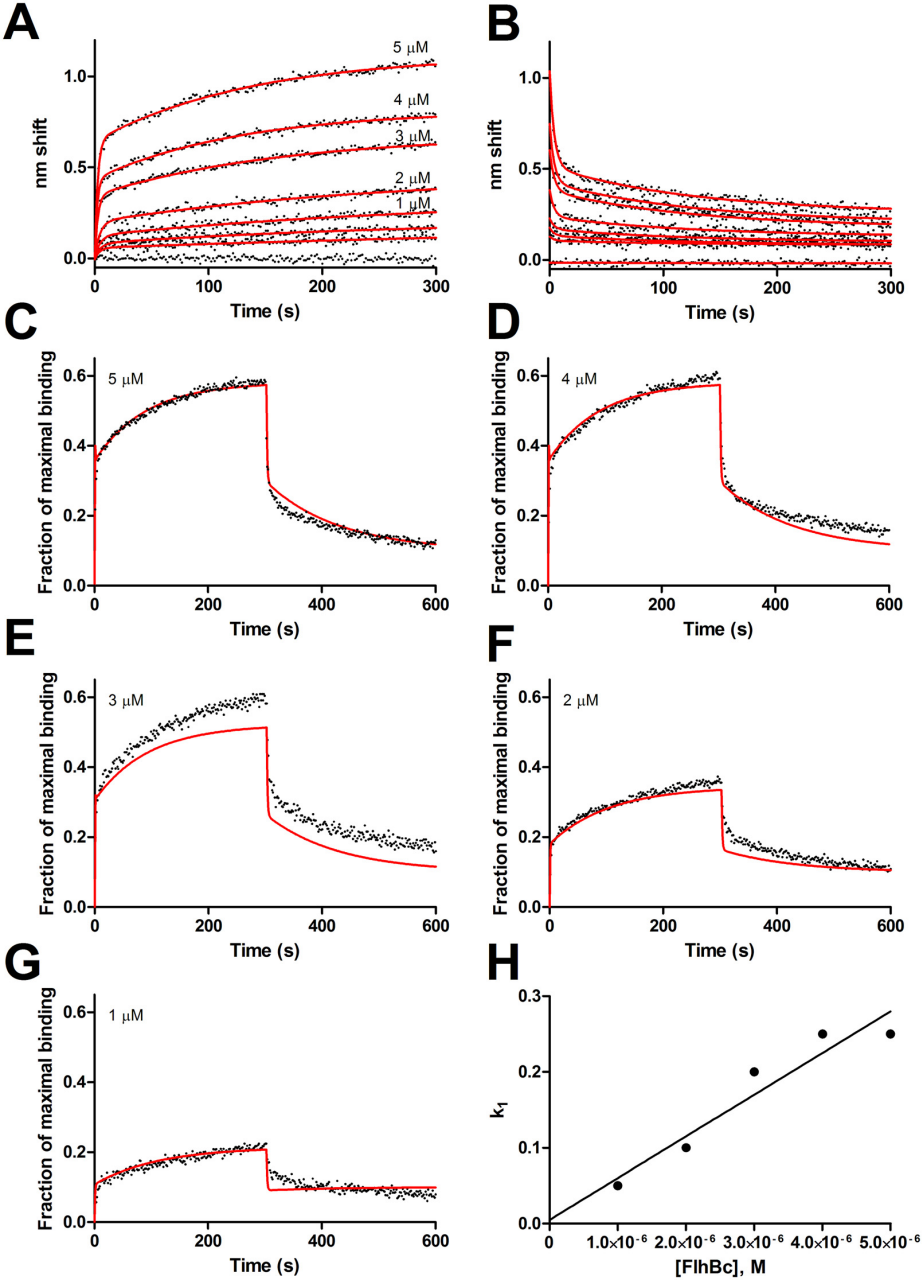
Simulation of FliK-FlhB_C_ binding. A and B, association and dissociation phases. Concentrations of FlhB_C_ were 5, 4, 3, 2, 1, 0.5, 0.25, and 0 μM. The 0.5 and 0.25 μM traces are unlabeled. Fits to individual two-state exponentials are shown as red lines. C-G, Simulations of the 5, 4, 3, 2 and 1 μM data with a conformational change model using global parameters for k_off_ and k_on_ and k_off_ for transition to the conformationally altered state (Table 2). H, Apparent k_on_ vs [FlhB_C_] to determine the global k_on_ (= slope).

We previously reported a K_D_ of 3.2 μM for FliK-FlhB_C_ binding determined by steady state analysis of SPR data [13]. Delving further into the complexity to better understand the kinetics, BLI sensorgrams were collected for a concentration course ranging from 0 to 5 μM FlhB_C_. As shown in Fig 2A and 2B, association and dissociation phases could be fit by two exponentials, i.e. parallel events, but there was no global solution that yielded constants that fit two independent states. Instead, numerical simulations were performed using differential equations constructed from a conformational change model. Simulations of association-then-dissociation are shown for 5, 4, 3, 2 and 1 μM in Fig 2C–2G. Parameters for constants used in the simulations are shown in Table 1 and include slow transitions to and from the AB* state. Plotting k_1_ determined from simulations, which is equivalent to the observed rate constant (k_obs_) for initial binding in that it also accounts for dissociation occurring during the association phase, vs. analyte concentration (Fig 2H) yielded k_on_ of 5.5 x 10^4^ M^−1^s^−1^. Combination with a k_off_ of 0.44 s^−1^ gave a K_D_ of 8.0 μM for the initial binding event, consistent with our earlier study. Supporting the conformational change model is the observation that the amplitude of the slow-off state in the dissociation phase varied proportionately with the length of the association phase (S2 Fig). It should be noted that amplitude variations between full kinetic characterizations and the Fig 1 survey are likely a function of different specific binding activities of different preparations for both ligand and analyte. All concentration courses in this experiment were done with dilutions of the same preparation. We also note that the overall K_D_ determined by steady state analysis, i.e. including the slow states, for FliK-FlhB_C_ in Fig 2 is 2.1 μM S3 Fig), almost identical to that of the preparations used in the earlier report despite very different amplitudes.

**Table 1:** Parameters determined by simulation of FliK-FlhB_C_ binding.

[_FlhBC_], μM	5	4	3	2	1
k_1_ (s^-1^)	0.25	0.25	0.2	0.1	0.05
k_2_ (s^-1^)	0.44	0.44	0.44	0.44	0.44
k_3_(s^-1^)	0.0085	0.0085	0.0085	0.0085	0.0085
k_4_(s^-1^)	0.008	0.008	0.008	0.008	0.008
B_max_	1.7549	1.7549	1.7549	1.7549	1.7549

**Fig 3:**
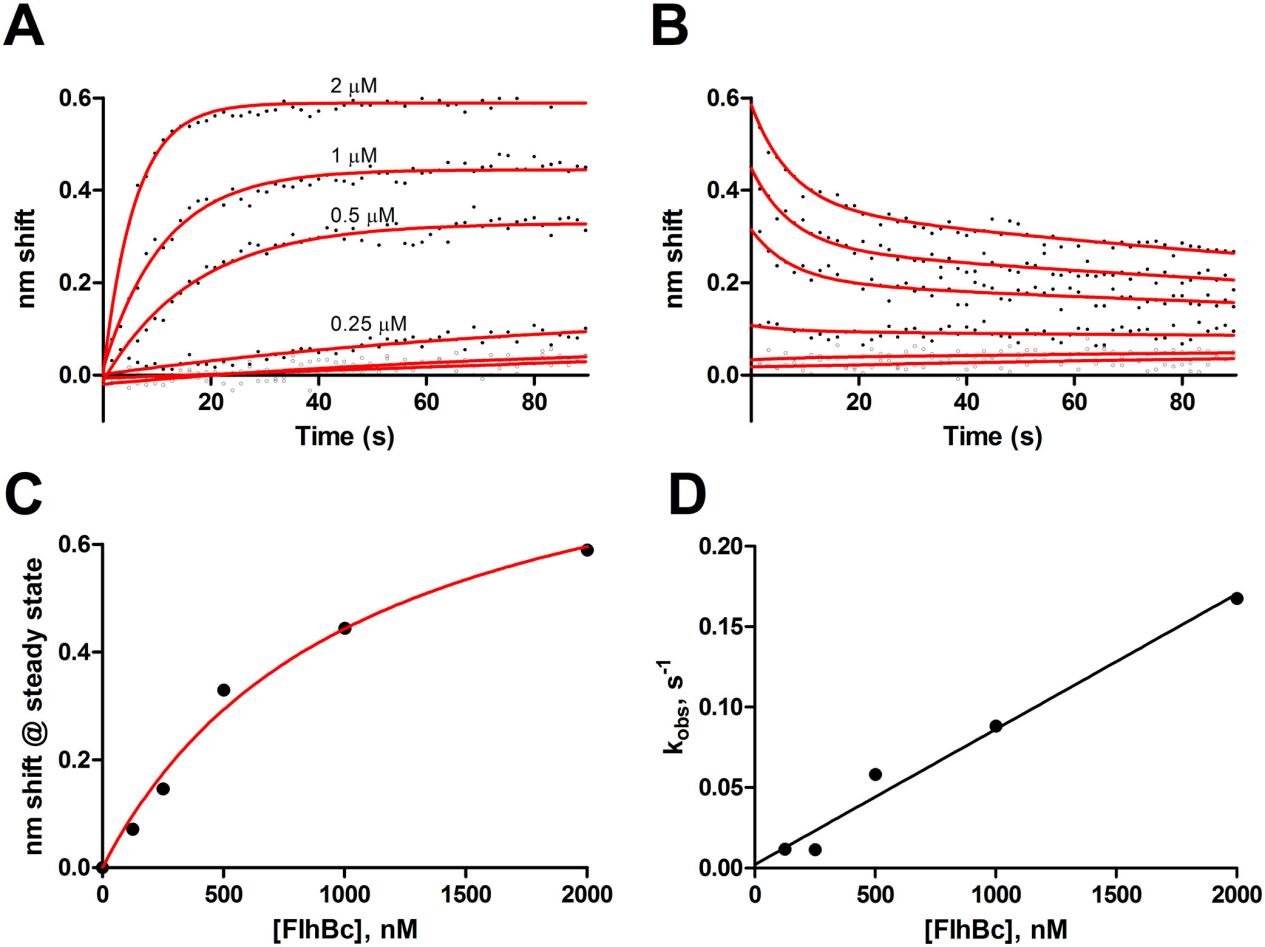
FlhA_C_-FlhB_C_ binding. Ligand FlhA_C_ was exposed to 2, 1, 0.5, 0.25 and 0.125 μM FlhB_C_. A, association with fits to a one-state model B, dissociation with fits to a global two-state model C, steady state analysis. D, k_obs_ vs. [FlhB_C_] to estimate kinetic constants, R^2^ = 0.98.

Ligand FlhA_C_-analyte FlhB_C_ binding also exhibited complexity and did not fit global one-state association-then-dissociation models. Single exponentials did fit the association phase (Fig 3A). Global two-state exponentials could fit dissociation with k_off_s of 0.13 s^−1^ and 4.7 x 10^−3^ s^−1^ (Fig 3B). Saturation analysis (Fig 3C) yielded a K_D_ of 1.1 μM. Plotting k_obs_ vs [FlhB_C_](Fig 3D) led to an estimate of k_on_ of 8.5 x 10^4^ M^−1^s^−1^ and thus a nominal one-state k_off_ of 0.09 s^−1^, though caution should accompany interpretation of these values (see Discussion).

**Fig 4:**
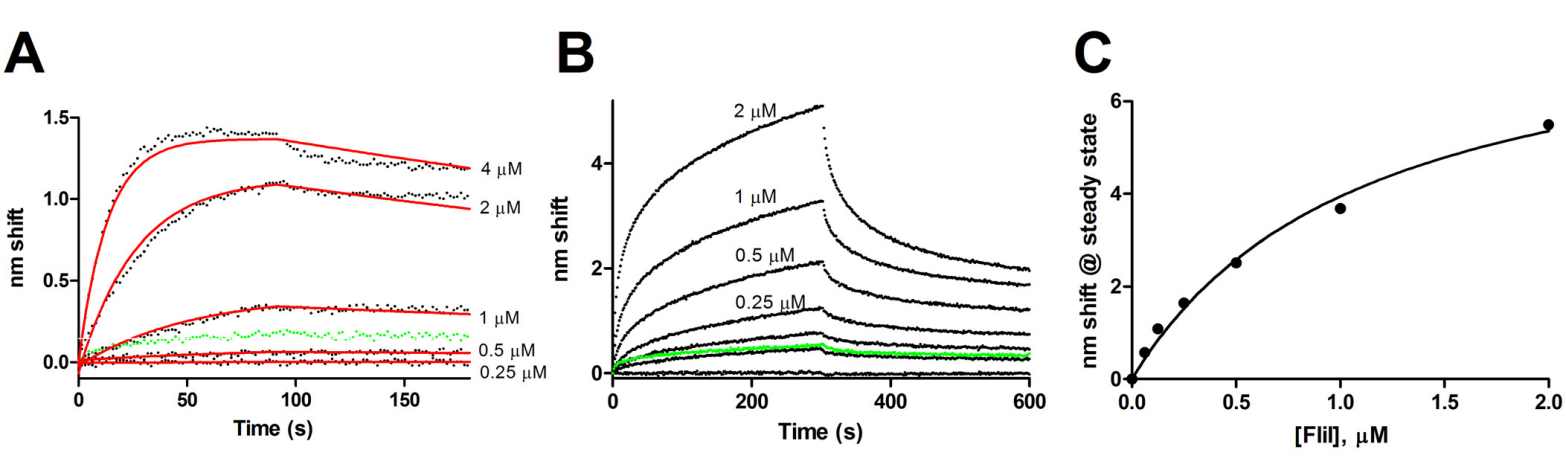
FlhB_C_-FliI binding. FlhB_C_ was used as ligand to examine binding of FliI in the absence and presence of ATP. A, Binding of 4, 2, 1, 0.5 and 0.25 μM FliI with fits to a global one-state association-then-dissociation model shown in red. The green points are 4μM FliI exposed to a sensor without FlhB_C_, e.g. NSB. B, FlhB_C_-FliI binding with FliI preequilibrated with 5 mM ATP and 5 mM MgCl_2_ added to all phases. FliI concentrations were 2, 1, 0.5, 0.25, 0.13, 0.062 and 0 μM. Green points represent the 2 uM sample binding to sensor without ligand. C, Steady state analydis of FliI with ATP from panel B, K_D_ = 1.1 μM.

FlhB binds FliI via its cytoplasmic domain. As shown in Fig 4, ligand FlhB_C_ bound FliI both in the absence and presence of Mg^2+^-ATP. For FliI without ATP (Fig 4A), fits to a global one-state model yielded a K_D_ of 84 nM with a k_on_ of 1.8 x 10^4^ M^−1^s^−1^ and k_off_ of 1.5 x 10^−3^ s^−1^. Preincubation of analyte FliI with an excess of ATP resulted in near elimination of nonspecific binding and more complex kinetics; substantial fast-on and fast off-states are seen relative to the no ATP sample. Data do not fit two-state models, perhaps reflecting additional states induced by oligomerization of FliI (see Discussion). K_D_ determined from steady state analysis (Fig 4C) is 1.1 μM.

### Full-length FlhB interactions

**Fig 5:**
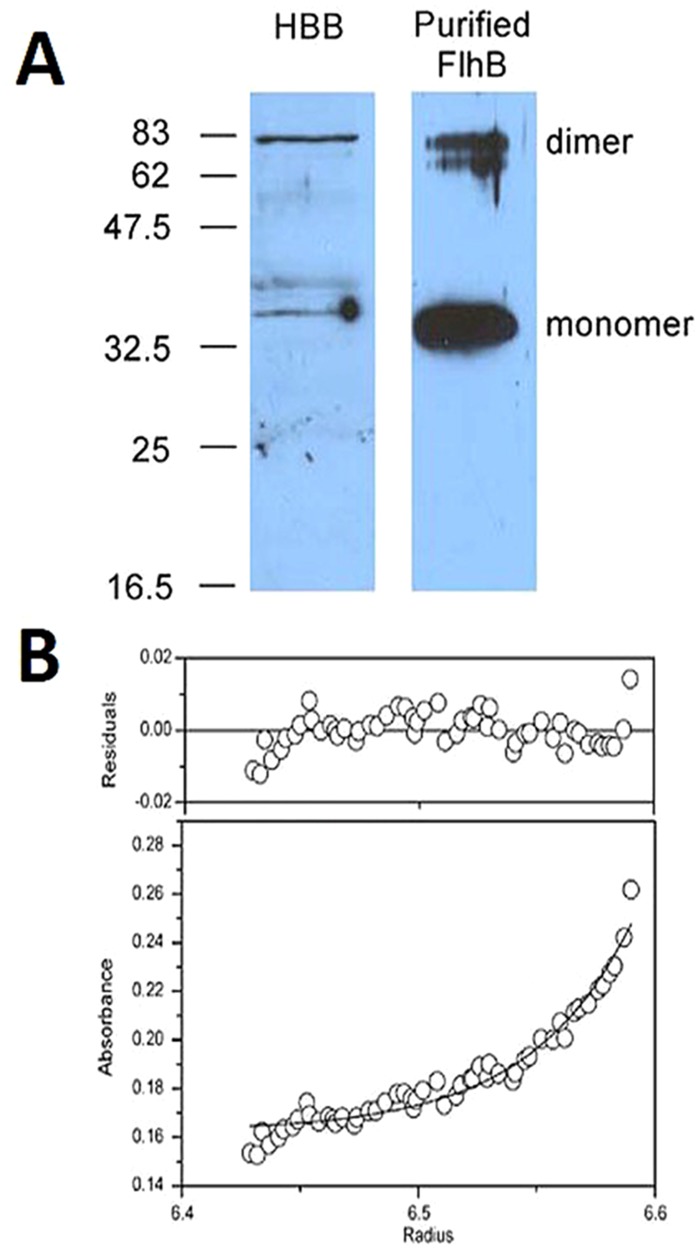
Full-length FlhB forms a dimer. A, anti-FlhB immunoblot of hook-basal body preparation (HBB) and purified FlhB(N269A). Approximate locations of molecular weight standards in kDa are shown at left. B, sedimentation equilibrium analytical centrifugation. A fit is shown to a single-species model, the molecular weight of which is 84.1 kDa (monomer of tagged FlhB(N269A) = 42.3 kDa).

FlhB_C_-FlhB_C_ interactions (Fig 1) were at best minimally observable, consistent with earlier studies that found questionable or no interaction [12,21]. We report here purification of solubilized FlhB under non-denaturing conditions using a procedure modified from a prior method used to purify FlhA [22]. The uncleavable but export competent N269A variant [14] was used to assure retention of the carboxyl-terminal subdomain in the solubilizing conditions used (though later purification of wild-type FlhB from pMM9, which complements a *flhB* null, showed that the subdomain consisting of residues 270–383 is retained (S1 Fig)). Anti-FlhB immunoblots of hook-basal bodies (HBBs) prepared from SJW880 [28] under conditions in which the C ring and export apparatus proteins are retained [29] (gift from Noreen R. Francis) demonstrated significant SDS-stable dimerization, as did purified FlhB(N269A) (Fig 5A). Full-length FlhB(N269A), solubilized in the neutrally buoyant, nondenaturing detergent C_8_E_5_, formed a stable dimer in a sedimentation equilibrium ultracenrifugation experiment (Fig 5B). Fits to a single species model produced a molecular weight of 84.1 kDa, consistent with a FlhB dimer. The tagged monomer is ~42.3 kDa.

**Fig 6:**
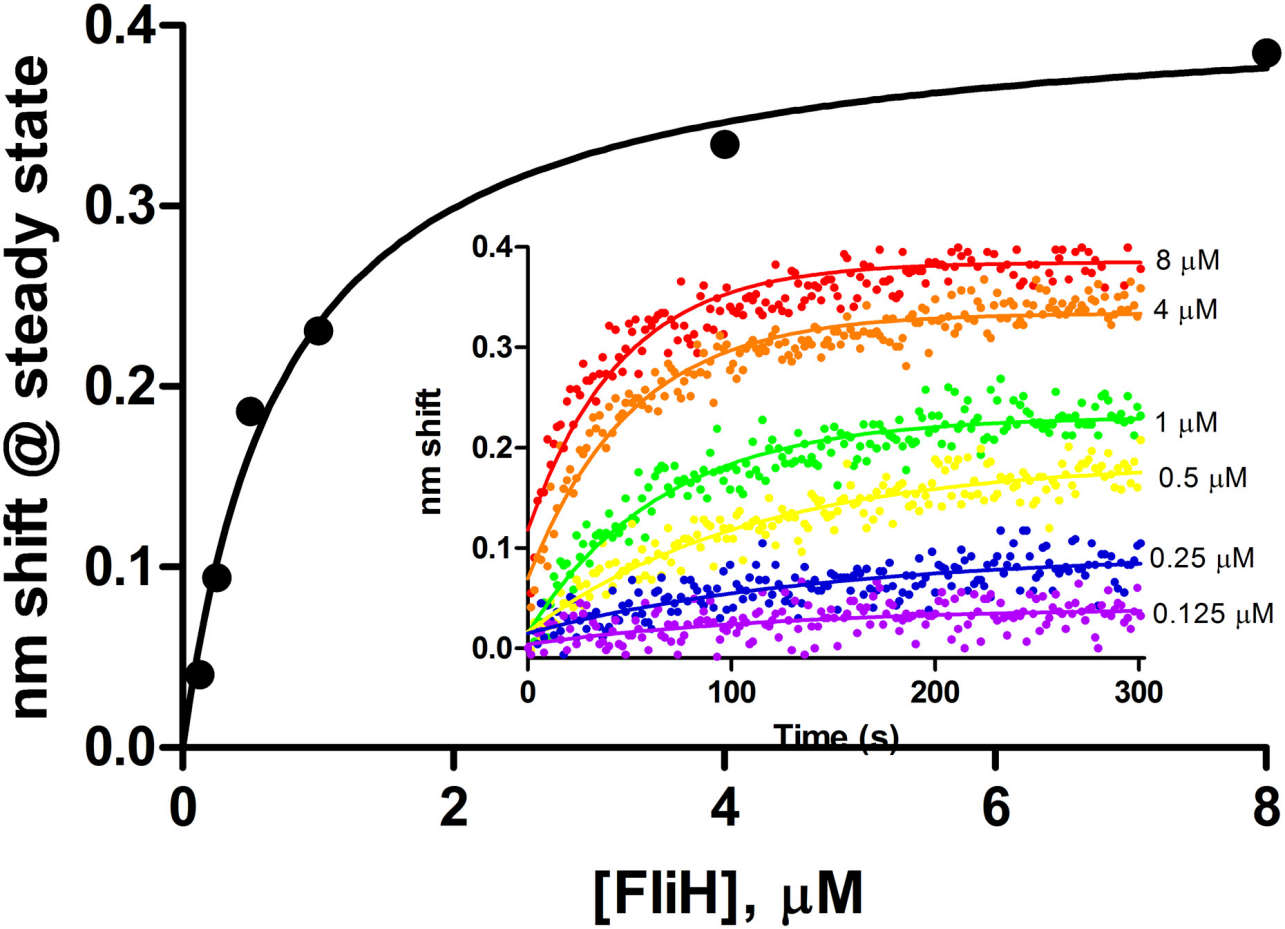
Steady state analysis of FliH binding to full length wild-type FlhB. Association phases from which steady state amplitudes were determined are shown in the inset. FliH concentrations ranged from 0.125 to 8 μM.

Purified wild-type FlhB exhibited specific binding to FliH as ligand. Dissociation anomalies perhaps due to detergent effects prevented kinetic analysis. K_D_ determined from steady state analysis (Fig 6) was 0.8 μM. The 2 μM sample was excluded from analysis due to anomalous readings from that channel, though its inclusion would render a K_D_ of 0.9 μM with a concomitant reduction in R^2^ from 0.99 to 0.82. Further experiments with other analytes were precluded by instability of the FlhB preparations; we hope to examine them in future studies.

In summary, the present results assign rate and affinity constants to binding of FlhB to several apparatus proteins and provide mechanistic clues for T3S. They are consistent with a conformational change in FlhB upon FliK binding and ATP-induced kinetic alterations in FlhB-FliI interactions as well as weak FlhB-FlhA cytoplasmic domain interactions and FliH interactions with full length FlhB.

## Discussion

Perhaps the best information about protein-protein interactions in the flagellar T3SS originates from intergenic suppression studies, e.g. [16,30]. However, much of the current conception of these interactions is based on qualitative equilibrium methods such as immunoaffinity blotting and copurification that can effectively identify only strong interactions [12,21,22,31]. These methods can also make interpretation difficult due to NSB. NSB was certainly a challenge in the present study and may be an inherent consequence of examining pairwise interactions between proteins whose normal environment is within a membrane-integrated macromolecular complex. We were able to measure NSB via the proxy of BSA binding, performing full analyses only when it was not a substantial fraction of total binding.

BLI of course has interpretative limitations as well. Since sensors are coated in ligand, sensors without ligands are not true references in that they can present a surface that differs in electrostatic and other properties. Use of BSA as a non-related ligand presents similar concerns, though binding of export proteins to both BSA and sensors without ligand was similar (data not shown). Ligand activity and presentation upon biotinylation could explain failure to observe binding on reversal of ligand-analyte pairings.

Interestingly, significant binding was not observed with several interactions previously reported. While present results cannot rule out interactions not observed, it is possible that at lower concentrations than those used in affinity blotting, FlhB engages in a different set of interactions. For example, ligand FlhB_C_ bound only FliI and perhaps FlhB_C_, exhibiting essentially no affinity for other apparatus proteins even though positive in affinity blotting [21]. Another possibility is that interactions may be biologically relevant even though they associate slowly but are stable once formed. The weaker interactions (FliK-FlhB_C_, FlhB_C_-FliI (with ATP), and FlhA_C_-FlhB_C_) are the ones more likely to undergo dynamic changes during export as they exhibit significant off rates.

All of the characterized interactions exhibited more than simple one-state binding. Whether the secondary events are biologically relevant is an open question. We suspect the initial, fast but low affinity events are the relevant ones and that the slower on and off events may be due to aggregation, surface-associated denaturation or other biologically irrelevant events. Surface-associated denaturation in BLI has been observed for calmodulin-nitric oxide synthase [32] and *Helicobacter pylori* UreE-UreG binding [33]. A potential example in this study is the slow-off FlhA_C_-FlhB_C_ dissociation phase (Fig 3B).

Kinetic and affinity constants determined with varying degrees of veracity are shown in Table 2.

**Table 2:** Kinetic constants determined from BLI experiments for binding to FlhB_C_.

Analyte	K_D_	k_on_ (M^−1^s^−1^)	k_off_ (s^−1^)
FlhA_C_	1.0 μM	8.5 x 10^4^	0.085
FliI (-ATP)	84 nM	1.8 x 10^4^	1.5 x 10^−3^
FliI (+ATP)	1.1 μM	ND	ND
FliK	8.0 μM	5.5 x 10^4^	0.44

ND, not determined. Constants are expressed with respect to the monomer concentrations.

### FliK-FlhB_C_ interactions

Though known to interact for many years, binding between FliK and FlhB_C_ was first directly observed using biosensing [13]. The one-state with conformational change model described herein fits observed FlhB-FliK binding. Affinity for the initial binding and dissociation is 8 μM, in good agreement with the previous report, which was determined by saturation binding and reflects both states. The fast-on, fast-off initial binding is consistent with intergenic suppression data and failure to observe interactions by equilibrium methods. Slow transitions to and from the conformationally altered state (AB*, see Results) render it a minor event, but one sufficient to explain the complexity observed. Whether it is a biologically relevant state and whether it changes in the presence of other proteins, e.g. substrates, or structural changes brought about by hook completion, remains a subject for further investigation, though conformational flexibility appears to be important for FlhB function [34]. Alterations in the dynamics of the conformational change would also be consistent with the temporal tape measure model.

### FlhA_C_-FlhB_C_ interactions

Compared to the NSB indicated by FlhB_C_ binding to BSA sensors (Fig 1B), FlhA_C_-FlhB_C_ binding exhibited fast on and fast off components (Fig 3). The most likely interpretation is that these proteins possess weak affinity for one another and that the slow-off state represents an irreversible, biologically irrelevant state. Conversely, it may signal a conformationally changed, high affinity state, but qualitative evidence suggesting weaker [21] or undetectable [22] binding supports the former interpretation. FlhA_C_-FlhB_C_ interactions are depicted as gating the membrane pore, e.g. [35]. Weak binding in the absence of the transmembrane domains may be exemplary of this.

### FlhB-FliI Binding

ATP induces hexamerization in FliI [36,37]. The present results show that in addition to inducing oligomerization, ATP alters FliI interactions with FlhB_C_. Presumably monomeric FliI without ATP shows tight binding to FlhB_C_ with relatively low NSB (Fig 4A). Addition of an excess of ATP resulted in substantially lower affinity (Fig 4C) but faster association and dissociation and additional complexity consistent with the hypothesis that FliI_6_ undergoes repeated binding and release events in delivering export-competent substrates to the export gate [38] and recent observations of FliI turnover in the basal body [39].

### FlhB dimerization and interaction with FliH

Ferris et al. extensively searched for FlhB_C_-FlhB_C_ interactions, finding none using equilibrium methods [12]. Our BLI data, too, indicate very little interaction between the cytoplasmic domains, though there may be some low affinity binding. Indeed, one interpretation of the complexity observed in BLI of FliK-FlhB_C_ binding was oligomerization of FlhB_C_ [13]. Development of a purification scheme for native, full-length FlhB allowed us to show that it forms a stable dimer in detergent micelles. Additional evidence from the HBBs suggests that FlhB forms dimers *in vivo* as well. The high affinity of the transmembrane domain-containing FlhB and the extremely low affinity (if present at all) of the cytoplasmic domains for themselves may hint at the dynamics of FlhB; the proximity of cytoplasmic domains forced by dimerization of the transmembrane domains may facilitate otherwise weak binding that may undergo cycles of association and dissociation as secretion occurs.

FliH exhibited significant binding only to full-length FlhB (Fig 6). Two possibilities suggest themselves: the binding site may reside at least partly within the transmembrane domain of FlhB; or FliH may be active with respect to FlhB binding in detergent micelles, which can be considered unsurprising as it partitions with the membrane even in the absence of basal bodies [40]. Other apparatus proteins were not investigated in this study with respect to binding the full-length FlhB due to the difficulty of the purification and the loss of FlhB binding activity over time. We hope to characterize these events in a future study.

The present results expand knowledge of the dynamic interactions of FlhB with other export apparatus proteins and assign rate an affinity constants to them. In short, FlhB stably dimerizes and stably binds FliH; FlhB_C_ binds FliK and FlhA_C_ with micromolar affinity and complex kinetics. Interactions with FliI shift upon addition of ATP, lowering affinity but increasing the rates of association and dissociation. The complexities observed underlie the mechanism of T3S. How interactions change when they are more than pairwise, as *in vivo*, is an active area of investigation.

## Supporting Information

S1 Fig Purification of full length FlhB. A, Coomassie stained SDS-PAGE of samples taken during purification. Lanes are: 1, uninduced cells; 2, induced cells; 3, crude lysate; 4, 1^st^ low-speed supernatant (clarified lysate); 5, 1^st^ high-speed supernatant; 6, 1^st^ high-speed pellet (crude membranes); 7, solubilization (overnight); 8, second high-speed pellet; 9, solubilized sample (load); 10, flow-through 11; pooled first wash; 12, final wash; E1-5, eluted protein fractions 1–5. B, Immunoblotting analysis of uninduced (U), induced (I) and purified (E2, diluted 10x), with positions of FlhB_TM+CN_ and FlhB_CC_ denoted with arrowheads at right. Note that anti-His only responds to FlhB_TM+CN_ as the His-tag is amino-terminal, anti-FLAG is overexposed and anti-FlhB is more reactive to FlhB_CC_, as has been noted previously (31).
(JPG)

S2 Fig Slow-off state increases in amplitude as a function of association time. Ligand FliK was exposed to analyte 2 μM FlhB_C_ for various times of association after which dissociation was monitored for 300 s. Time of association was 10 s (green), 30 s (yellow), 60 s (orange), 180 s (blue), and 900 s (brown).
(TIF)

S3 Fig Slow-off state increases in amplitude as a function of association time. Ligand FliK was exposed to analyte 2 μM FlhB_C_ for various times of association after which dissociation was monitored for 300 s. Time of association was 10 s (green), 30 s (yellow), 60 s (orange), 180 s (blue), and 900 s (brown).
(TIF)

S1 Table Plasmids used in this study. (PDF)
