# Supplementary figures and images for: Weak Interactions between Salmonella enterica FlhB and Other Flagellar Export Apparatus Proteins Govern Type III Secretion Dynamics
Source: PLoS One. 2015 Aug 5;10(8):e0134884. doi: 10.1371/journal.pone.0134884 (PMC4526367; doi:10.1371/journal.pone.0134884)

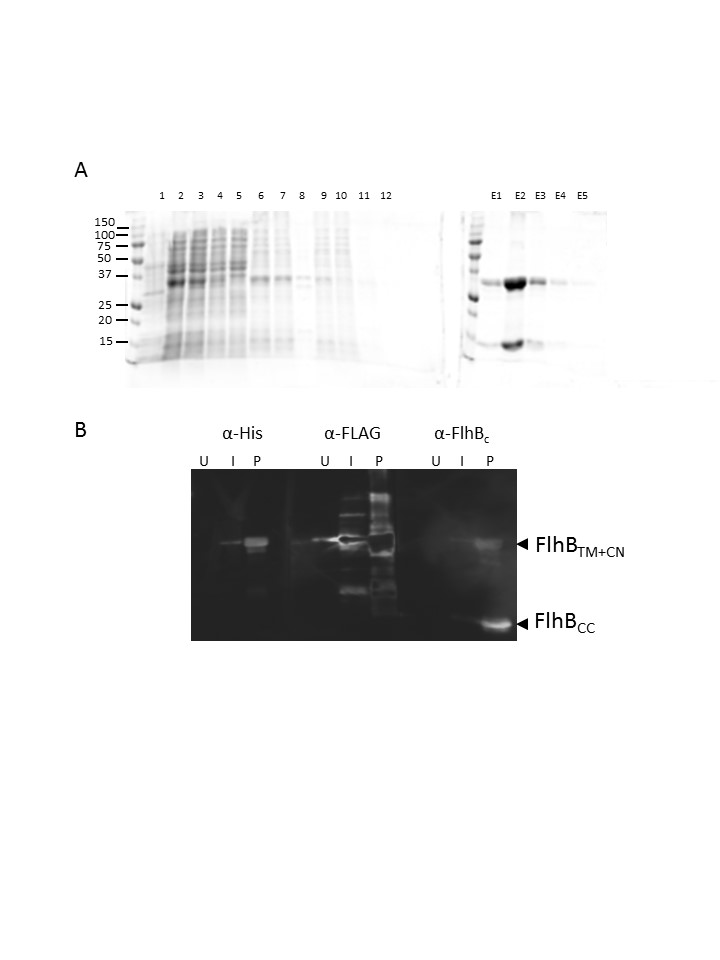

Supplement: S1 Fig — A, Coomassie stained SDS-PAGE of samples taken during purification. Lanes are: 1, uninduced cells; 2, induced cells; 3, crude lysate; 4, 1st low-speed supernatant (clarified lysate); 5, 1st high-speed supernatant; 6, 1st high-speed pellet (crude membranes); 7, solubilization (overnight); 8, second high-speed pellet; 9, solubilized sample (load); 10, flow-through 11; pooled first wash; 12, final wash; E1-5, eluted protein fractions 1–5. B, Immunoblotting analysis of uninduced (U), induced (I) and purified (E2, diluted 10x), with positions of FlhBTM+CN and FlhBCC denoted with arrowheads at right. Note that anti-His only responds to FlhBTM+CN as the His-tag is amino-terminal, anti-FLAG is overexposed and anti-FlhB is more reactive to FlhBCC, as has been noted previously (31). (JPG) [file pone.0134884.s001.jpg]
