# Supplementary material for: Weak Interactions between Salmonella enterica FlhB and Other Flagellar Export Apparatus Proteins Govern Type III Secretion Dynamics
Source: PLoS One. 2015 Aug 5;10(8):e0134884. doi: 10.1371/journal.pone.0134884 (PMC4526367; doi:10.1371/journal.pone.0134884)
Supplement: S1 Table — (PDF) [file pone.0134884.s004.pdf]

Plasmid	Brief Description	Reference
pGFB N269A	pET19b/His-FlhB(N269A)	14
pGFB$_c$	pET22b/FlhB$_c$-His	14
pMM9	pTrc99A/His-FLAG FlhB	This study
pMM 104	pET19b/His-FlhA$_c$	21
pMM 310	pet19b/His-FliH	21
pMM 1701	pET19b/His-FliI	21
pMM 405	pET19b/His-FliJ	21
pMM 850	pET19b/His-FliK	T. Minamino, unpublished
